# Comparative Study of Water-Leaching and Acid-Leaching Pretreatment on the Thermal Stability and Reactivity of Biomass Silica for Viability as a Pozzolanic Additive in Cement

**DOI:** 10.3390/ma11091697

**Published:** 2018-09-12

**Authors:** Weiting Xu, Jiangxiong Wei, Jiajian Chen, Bin Zhang, Peng Xu, Jie Ren, Qijun Yu

**Affiliations:** 1 School of Materials Science and Engineering, South China University of Technology, Guangzhou 510641, China; mszhangbin@mail.scut.edu.cn (B.Z.); concyuq@scut.edu.cn (Q.Y.); 2 Department of Civil Engineering, Foshan University, Foshan 528000, China; chenjiajian@fosu.edu.cn; 3 Department of Mechanics and Civil Engineering, Jinan University, Guangzhou 510632, China; t60at3@gmail.com; 4 Department of Infrastructure Engineering, The University of Melbourne, Melbourne, VIC 3010, Australia; renjie630@gmail.com

**Keywords:** amorphous silica, crystallization sensitivity, water-leaching pretreatment, rice husk ash, cement

## Abstract

The present work aims to introduce a novel and eco-friendly method, i.e., a water-leaching pretreatment for extracting highly reactive biomass silica from rice husk (RH), for viability as a pozzolanic additive in cement. For comparison, the traditional acid pretreatment method was also employed throughout the experimental study. The silica from RH was extracted using boiled deionized water and acid solution as leaching agents to remove the alkali metal impurities, and then dried and submitted to pyrolysis treatment. The results indicated that potassium was found to be the major contaminant metal inducing the formation of undesirable black carbon particles and the decrease in crystallization temperature of amorphous RHA silica. The boiling-water-leaching pretreatment and acid-leaching pretreatment on RHs significantly removed the metallic impurities and reduced the crystallization sensitivity of RHA silica to calcination temperature. A highly reactive amorphous silica with purity of 96% was obtained from RH via 1 N hydrochloric acid leaching followed by controlled calcination at 600 °C for 2 h. The acid treatments increased the crystallization temperature of silica to 1200 °C and retained the amorphous state of silica for 2.5 h. In the case of water-leaching pretreatment, leaching duration for 2.5 h could yield an amorphous silica with purity of 94% and render the silica amorphous at 900 °C for 7 h. The RHA silica yielded by water-leaching pretreatment presented a comparable enhancing effect to that of acid leaching on hydration and improved the strength of cement. Furthermore, compared with the acid-leaching method, the water-leaching pretreatment method is more environmentally friendly and easier to operate, and hence more widely available.

## 1. Introduction

Rice husk (RH) is the outer shell of the rice grain, which is a by-product of the rice milling process. It is an agricultural waste in all rice-producing countries. Most of the RH usually ends up either being dumped or burned in open spaces, which not only occupies a large land area but also represents a major source of contamination [1,2,3].

The major components of RH are organic materials such as hemicellulose, cellulose, and lignin, totaling about 85%, and the remaining ash content is 15–20% [4]. Of all the residues of edible plants, the ash obtained from the calcined RH has the highest silica content [5]. The orthosilicic acid was ingested from soil and groundwater by the rice crop and further polymerized in the tissue structure of the plant, contributing to the formation of amorphous silica in the husk. After burning, RH becomes rice husk ash (RHA), which normally contains 85–95% of silica (SiO_2_), 5–8% of alkali metal oxides, and some carbonaceous materials by mass [6].

Amorphous silica has a wide range of industrial applications, such as raw materials for ceramics synthesis, refractories, plastics, silica gels, silica chip, activated carbon and silica, catalysts, zeolites, ingredients for lithium-ion batteries, graphene, energy storage/capacitor, carbon capture, and drug delivery vehicles [7,8,9,10,11,12,13,14,15,16,17]. Compared with other industrial fields, the use of amorphous RHA silica in the production of concrete can realize large-scale consumption and reuse of waste, and hence eventually reduces the environmental impact due to improper disposal and land occupation. RHA is economical, widely available and highly reactive as a supplementary cementing material, due to its excellent physically filling and chemically pozzolanic effects [18,19,20,21,22]. It is also a promising substitute for replacing silica fume (SF) in high-performance concrete production.

Under a controlled calcination procedure and temperature, the calcination of RH removes hydrocarbon compounds and yields amorphous silica-rich powders with a large surface area. A highly reactive RHA silica is produced by burning RH at a temperature of 500 °C or lower for a comparatively protracted time under oxidizing environment or for a shorter time at a temperature of up to 700 °C [23]. The calcination of RH beyond this temperature may lead to the conversion of amorphous silica to crystalline silica polymorphs (quartz, cristobalite, and tridymite) [24]. Moreover, these crystals tend to predominate in unacceptable quantities and particle agglomeration may occur as the temperature increases, thereby reducing the chemical reactivity of RHA silica [25].

The temperature-sensitive nature of RHA silica leads to a rigid requirement for combustion instrument and pyrolysis technology, consequently capping the large-scale industrial production. To achieve amorphous silica ash of a desirable quality, a controlled combustion temperature and time are compulsory. Moreover, even if the RH is burned under controlled calcination conditions, the internal heat of the RH heap is difficult to release, and the temperature will rapidly exceed the crystallization point of the RHA silica. Thus, reducing the crystallization sensitivity of RHA silica to calcination temperature is the most critical issue for achieving large-scale production of biomass silica.

Studies indicate that the phase transformation temperature of RHA silica is markedly influenced by the inorganic chemical impurities [26]. Alkali metals, such as potassium, sodium, and calcium salts in RH, preferably react with silica to form eutectic mixtures with low melting points. At a high concentration of potassium or sodium, the melting point of eutectic mixtures is as low as 600–700 °C, significantly reducing the normal crystalline temperature of RHA silica [27].

The acid-leaching pretreatment on RH has been proved to be effective to remove the metallic contamination. The amorphous silica obtained from the acid pretreatment contains very high purity silica (above 95%) [28,29]. However, the use of acid as a leaching agent usually causes corrosion in pipes and instruments, and increases the operational difficulty and production costs. Hence, it is of great importance to find a novel eco-friendly pretreatment method to remove metallic impurities from RHA silica.

In addition, the kinetics of the thermal stability of amorphous RHA silica under various leaching‒pyrolysis treatments have not been clearly established. Moreover, major metallic contamination, which induces the amorphous silica in biomass RH compounds to crystallize at a relatively low calcination temperature, has not been verified.

In view of the fact that the biomass metallic salts are mostly soluble, this study explores using boiling water as a leaching argent for rinsing off alkali metal impurities in RHA silica and consequently obtaining a purified and highly reactive RHA silica. The present work aims to reveal the influence of water-leaching pretreatments on the crystallization behavior and chemical reactivity of RHA silica, consequently extending our knowledge for the industrialized mass manufacture and application of biomass silica. For comparison, the traditional acid pretreatment method is also assessed throughout the study. In this study, the effects of boiling-water-leaching pretreatment and acid-leaching pretreatment on crystallization sensitivity of RHA silica to pyrolysis conditions (calcination time and duration) were investigated and compared. The influences of metallic impurity and content on the thermal stability of RHA silica were identified by testing the crystallization behavior of the mixtures of purified RH and alkali salt. To examine the pozzolanic reactivity of the as-prepared RHA silica samples for their viability as a pozzolanic additive for the production of high-strength cement and concrete, the compressive strength, chemically bound water, and hydration product mineralogy of cement pastes with 10% cement replaced by RHA samples were examined.

## 2. Materials and Methods 

### 2.1. Materials

Rice husk (RH) collected from a local rice processing plant (Academy of Agricultural Sciences Institute, Guangzhou, China) during the process of rice manufacturing was used as the starting material in this study. Commercially available chemical reagents, such as hydrochloric acid (HCl), sulfuric acid (H_2_SO_4_), and nitric acid (HNO_3_), were of analytical grades and used as the acid-leaching agents for RH. Deionized water was used for the boiling-water-leaching pretreatment test as well as the residue rinsing of RH throughout the experiments.

### 2.2. Leaching‒Pyrolysis Treatment of RH

RH samples were examined in this study by employing the leaching‒pyrolysis steps. Two leaching pretreatment regimes, i.e., the boiling-water-leaching and acid-leaching pretreatment on RH, were applied in this study to remove alkali metal impurities from RH.

In the case of boiling-water-leaching pretreatment, the RHs were weighed into three consignments of 10 g each. Each consignment was soaked in boiling water of 100 °C for 2.5, 5, or 10 h, and then filtered and air-dried.

As for the acid pretreatment, RHs were weighed separately in three batches of 10 g each, and each batch was subjected to immersion in the acid solution (hydrochloric acid, sulfuric acid, or nitric acid) at a concentration of 1, 2, or 3 N acid solution for 1 or 2.5 h with constant stirring at ambient temperature. After the acidic solution was drained off, RH was rinsed with deionized water until the pH rose to 7, then filtered and air-dried.

After the leaching pretreatments, the as-pretreated RH samples were collected and dried in a drying oven at 50 °C for 24 h, and then separately subjected to pyrolysis in an electronic furnace for burning out at the desired temperature (600–1200 °C) until the appointed time (0.25–2 h). After the furnace hearth naturally cooled down to room temperature, the ash obtained was collected and subjected to grinding for 10 min in a laboratory mill. The ground ashes were kept separately in a desiccator for future material characterization tests.

The original RH was represented by RH. In the case of acid-leached RH, the HCl-, H_2_SO_4_-, and HNO_3_-pretreated RH samples were marked as Cl-RH, S-RH, and N-RH, respectively. The boiling-water-leaching-pretreated RH samples were labeled W-RH. Accordingly, each RHA sample is named for clarification, e.g., 1Cl-RHA600-2h, which implies RHA with HCl leaching pretreatment for 1 h and then burning out at 600 °C for 2 h.

### 2.3. Testing of RH

Scanning electron microscopy (SEM) analyses were conducted to study the structure of acid-leaching- and water-leaching-pretreated RH samples at the micro level. The dried RH samples were coated with gold in a sputter coater. The SEM experiment was performed on a PHILIPS ESEM XL-30 (FEI, Hillsboro, OR, USA) operating at 20 kV with a 15 mm working distance.

The functional groups in the RH samples were determined using an FTIR equipment BRUKER EQUINOX 55 (Bruker, Karlsruhe, Germany). The spectra were recorded with 32 scans at a resolution of 4 cm^−1^ in the range of 4000–400 cm^−1^.

### 2.4. Testing of RHA

#### 2.4.1. Characterization of RHA

Quantitative chemical analyses of RHA were accomplished by X-ray fluorescence. The particle size was determined by the laser diffraction analyzer Easysize 20 (OMEC, Zhuhai, China). The surface area and pore volume of RHA were measured by Brunauer‒Emmett‒Teller (BET) and Barrett‒Joyner‒Halenda (BJH) methods, respectively, according to ASTM D3663-03 using Micromeritics Tristar 3000 Surface Area (Micromeritics, Norcross, GA, USA) and Porosity Analyzer (Micromeritics, Norcross, GA, USA).

The functional groups in the RHA samples were detected by the FTIR technique.

The pozzolanic reactivity of RHA silica was examined by the electrical conductivity change test, i.e., the reaction between RHA and a saturated calcium hydroxide solution. Each test utilized 200 mL of solution, which contained approximately 0.4 g of Ca(OH)_2_. Solutions were placed in a plastic Erlenmeyer flask and stirred at a constant temperature of 80 °C. Initial conductivity values were registered by a conductivity meter. Then, 5 g of RHA was added. SiO_2_ reacting with Ca^2+^ ions led to the formation of C‒S‒H, a non-conductive compound, and hence decreased the solution conductivity over time. The conductivity change value was recorded as an indication of the chemical reactivity of amorphous silica.

X-ray diffraction (XRD, Bruker, Karlsruhe, Germany) patterns were obtained using a Bruker MSAL XD2 X-ray Diffractometer using CuKα operated at 36 kV and 24 mA. The scanning two theta is between 10° and 60°. EVA™ Software (Bruker, Karlsruhe, Germany) was used to record and analyze the structural pattern of the sample.

#### 2.4.2. Identification Tests for Metallic Contamination in RHA

To identify the metallic trace element that leads to the decrease in crystallization temperature of RHA silica composites, the potential contaminant elements (the constituent trace elements of RH) in terms of chlorine salts were separately incorporated into the HCl-purified RH. The trace element was incorporated into RH powder at a dosage of 2% by weight of the binder. The RH powder and chlorine salt were mixed for 1 min to obtain a homogenous powder mix. Then the mix was subjected to calcination at 600 °C for 2 h to yield an ash sample (silica composite).

To reveal the effect of potassium content on the crystalline behavior of silica in RHA, potassium in the form of KCl was incorporated into the acid-pretreated RH powder at five dosage levels of 0%, 0.5%, 1%, 5%, and 10% by weight of the binder. The binder of KCl and purified RH was mixed for 1 min to obtain a homogenous powder mix. Then the mix was subjected to calcination at 500 and 700 °C for 2 h.

All the ash samples obtained were labeled and sealed separately in plastic bags for XRD characterization tests.

### 2.5. Compressive Strength of RHA Incorporated Cement Paste

The compressive strength of 20 mm mortar cube after three, seven, and 28 days of moist curing was determined in accordance with the ASTM C 311-07 standard. This test was carried out to examine the pozzolanic activity of the ash samples with various leaching pretreatments. One control mix (P0) and five pastes ((PR, P2.5WR, P5WR, P10WR and PClR)) incorporating RHA obtained by calcining unpretreated RH or leaching-pretreated RH at 600 °C for 2 h were prepared. PR is the paste incorporating RHA yielded by calcining unpretreated RH at 600 °C for 2 h. P2.5WR, P5WR and P10WR represent the paste incorporating RHA obtained by 2.5, 5, and 10 h boiling-water-leaching pretreatment and calcining at 600 °C for 2 h, respectively; PClR denotes the paste incorporating RHA obtained by 1 h HCl leaching pretreatment and calcining at 600 °C for 2 h. All the pastes were blended with 10% RHA by mass of cement and had a water binder ratio of 0.4. The test yielded paste indicates the pozzolanic reactivity degree of RHA silica.

### 2.6. Chemically Bound Water Content of RHA Incorporated Cement Paste

To investigate the effect of the RHA silica on the hydration of cement matrix, a chemically bound water content test was conducted. After the compressive strength test, the broken paste fragments were selected and immediately immersed in anhydrous ethanol to stop cement hydration at the predetermined age. Then the fragments were baked in an electronic furnace at 105 °C for 16 h to a constant weight, and then naturally cooled down to the ambient temperature and weighed. After that the fragments were subjected to heating at 1000 °C for 20 min, and then cooled down and weighed. Each test was repeated three times to ensure accuracy. The data recorded were subsequently used to calculate the chemically bound water content in the paste samples.

### 2.7. XRD Analysis of Hydration Products of RHA Incorporated Cement Paste

To investigate the effect of RHA on the hydration products of cement, the XRD analysis of cement pastes without and with water-leaching and acid-leaching-pretreated RHA were determined at the age of 7 days. The XRD scanning were performed at two theta between 10° to 60°.

## 3. Results and Discussion

### 3.1. Chemical Compositions and Physical Properties of RHA

Chemical compositions and physical properties of RHAs without and with pretreatment are summarized in Table 1 and Table 2, respectively. It is seen that silica oxide forms the main component (89–96%) of RHAs with trace elements in the form of composite oxides K_2_O, Na_2_O, CaO, MgO, Fe_2_O_3_, and Al_2_O_3_. In the case of unpretreated RHA, 89.6% of silica was produced by calcining RH at 600 °C for 2 h. Potassium (K) was detected to account for the highest concentration (2.53%) among the metallic trace elements. With the increase in calcination temperature and duration, silica content and undesirable carbon residue (represented by LOI) were slightly increased. The specific surface area tends to decrease with the increase of calcination temperature and duration, contributing to the particle agglomeration. It is apparent that the capacity of controlled calcination condition for improving the silica purity and chemical reactivity of silica is rather limited.

The leaching pretreatment process results in significant changes in trace element concentrations in RHA. In the case of acid-leaching pretreatment, HCl exerts superior performance in removing metallic impurities to the other leaching agents. The RHA yielded by 1 N HCl leaching pretreatment and calcination at 600 °C for 2 h possesses the highest silica content (96.41%) and surface area (248.21 m^2^/g). The trace elements are reduced to a very low level, especially in the case of potassium, which is reduced to 0.06%. HNO_3_ and H_2_SO_4_ as leaching agents also have excellent performance in washing off alkali metals and improving the surface area, as well as reducing the carbon residue content of RHA.

In the case of boiling-water-leaching pretreatment, the RH pretreated by boiling water leaching for 2.5 h and burning out at 600 °C for 2 h yields RHA with a silica content of 94.03% and a surface area of 130.82 m^2^/g. However, extending the water-leaching duration to 10 h does not seem like a responsible way to increase the concentration and surface area of silica. Thus, a water-leaching duration of 2.5 h is sufficient for pretreating RH to extract pure silica. It is worth noting that, although the boiling water-leaching pretreatment is inferior in extracting a high content of silica and rinsing off metallic impurities in RH during the acid-leaching pretreatment, the boiling water-leaching pretreatment method is more eco-friendly and economical.

The weight loss data obtained from thermogravimetric studies are presented in Table 1. It is clearly seen that the residual organics or undesirable carbon of RHA are gotten rid of with greater ease from the acid-leaching- or water-leaching-pretreated RH upon heating in comparison to that of the unpretreated RHA.

The average particle size, BET surface area, pore volume, and pore diameter of RHA samples are given in Table 2. It is seen that leaching pretreatment significantly affects the surface area as well as the pore volume of RHA silica. The average pore diameter of acid-leached RHA, water-leached RHA, and unpretreated RHA is around 6, 6 and 32 nm, respectively, indicating that the RHAs produced are mainly mesoporous. The pore volume of the acid-leached RHA and the water-leached RHA are higher than that of the unpretreated one. It is noteworthy that either the acid-leaching pretreatment or the water-leaching pretreatment can markedly increase the surface area and the pore volume, as well as reduce the pore diameter of RHA. This is mainly attributed to the hydrolysis of lignin and cellulose into smaller compounds and the dissolution of alkali metals after leaching pretreatment, which promote the volatilization of fixed carbon in RHA with greater ease during combustion. Moreover, the removal of the organic carbohydrates causes the inside pores in RHA to open and hence leads to a more loose and highly porous structure of RHA.

**Figure 1:**
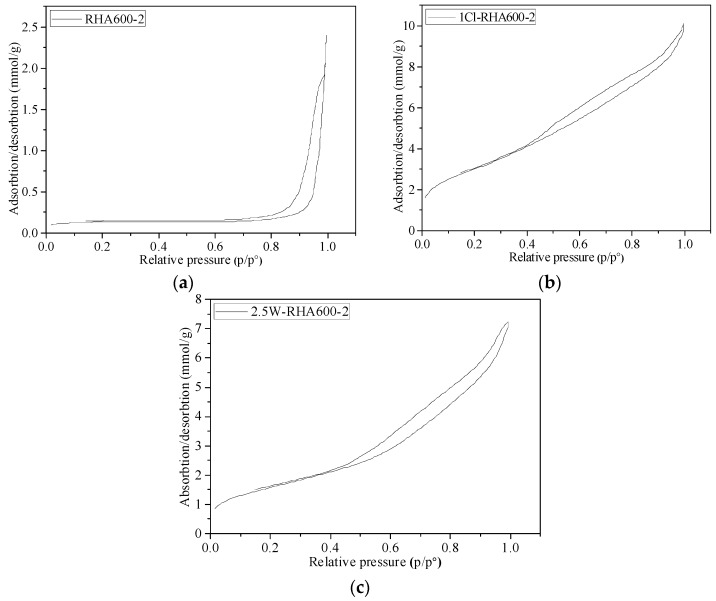
Nitrogen adsorption‒desorption isotherm for (**a**) untreated RHA; (**b**) acid-treated RHA and (**c**) water-treated RHA.

The N_2_ adsorption‒desorption isotherms and pore size distribution curves of the RHA samples are shown in Figure 1, respectively. It is seen that the adsorption is not limited to high values of P/P0 above 0.95. In addition, the isotherms present a vertical asymptotic profile at high values of P/P0, which is characteristic of a mesoporous structure with non-uniform sized particles.

It is seen from Figure 1a that the unpretreated RHA presented the type I hysteresis loop [30] according to de Boer’s classification of hysteresis loops, suggesting an intergranular pore structure formed by the spherical granules compactly accumulating together. As shown in Figure 1b,c, both the boiling water-pretreated RHA and the acid-pretreated RHA present type III hysteresis loops, indicating a bottle-shaped pore structure. The absorption and desorption of type III isotherms are more steep, suggesting that the porosity of the pretreated RHA is higher than that of the unpretreated one.

**Figure 2:**
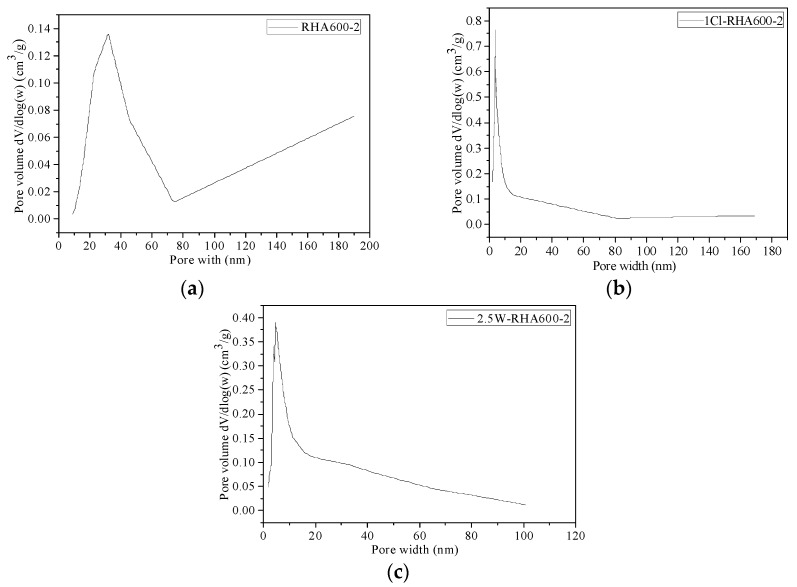
Pore size distribution calculated via BJH algorithm of (**a**) untreated RHA; (**b**) acid-treated RHA and (**c**) water-treated RHA.

Figure 2 gives the information on the BJH pore volume and diameter of RHAs. It is seen from Figure 2a that the unpretreated RHA has a mean pore diameter of 32.7804 nm, indicating that it is between mesoporous and macroporous. Its pore volume is 0.082997 cm^3^/g, suggesting a low quantity of pores. In contrast, the acid-leaching and water-leaching pretreatment lead to a reduction of RHA pore diameter to 6.4479 and 5.1521 nm, and an increase of pore volume to 0.257617 and 0.368958 cm^3^/g, respectively. It is evident that leaching pretreatment improves the internal porosity of RHA.

### 3.2. Conductivity Change Test of RHA

The conductivity change testing results are listed in Table 2. The conductivity change value is an indication of the pozzolanic reactivity of the testing powder. The greater the change value in conductivity of the testing solution, the higher the chemical reactivity of RHA silica. The conductivity change value of RHA silica shows both leaching pretreatment- and pyrolysis condition- (temperature and duration) dependent behavior. In the case of unpretreated ash RHA600-2, the conductivity change value is 0.38. In the case of acid-leaching-pretreated RHA samples, the conductivity change value of 1Cl-RHA600-2, 1N-RHA600-2 and 1S-RHA600-2 is 6.09, 5.91, and 5.73, which is 1502.6%, 1407.9%, and 1243.2% higher, respectively, than that of the unpretreated ash RHA600-2. In the case of water-leaching-pretreated RHA samples, the conductivity change value of 2.5W-RHA600-2 and 2.5W-RHA900-2 is 4.42 and 4.64, which is 1063.2% and 1121.1% higher, respectively, than that of RHA600-2. It is apparent that with the assistance of an acid- or water-leaching process, the metallic concentration in RH is significantly reduced, while the chemical reactivity of the yielded RHA is distinctly increased as compared to the unpretreated one. In addition, it is noteworthy that prolonging the duration of water leaching does not lead to an obvious improvement in the conductivity change value of the testing solution. In other words, excessive water-leaching pretreatment time is not related to a significant increase in the pozzolanic reactivity of RHA. Thus, a duration of 2.5 h for boiling water leaching is reasonable and economical.

### 3.3. FTIR Analysis of RH and RHA

**Figure 3:**
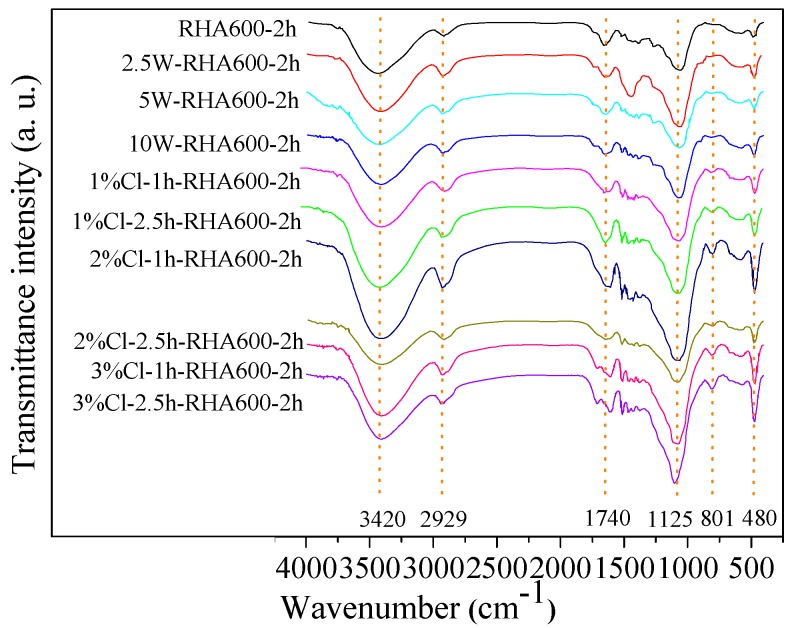
FTIR spectra of RH with and without various pretreatments.

The FTIR spectra of the unpretreated, HCl-leaching-pretreated, and water-leaching-pretreated RHs are shown in Figure 3. The broad band observed around 3420 cm^−1^ is attributed to the stretching vibration of C‒H and OH groups, indicating the cellulose structure and water absorption (hydroxyl groups bound to the cellulose structure) in RH [31,32]. The band at 2929 cm^−1^ represents the stretching and/or vibration of the C‒H group, attributed to the aliphatic-saturated compounds in the cellulose [33]. A slight shoulder at 1740 cm^−1^ is referent to the stretching vibration of the C=O bond in hemicellulose or carboxylic acid groups in the ferulic and *p*-coumaric components of the lignin [31,34]. These organic groups suggest that the cellulose and lignin are not rinsed off from RH by the acid-leaching or water-leaching pretreatment. The characteristic absorption peaks of SiO_2_ appear at 480 cm^−1^ (bending vibration of O‒Si‒O), 800–820 cm^−1^ (stretching vibration of Si‒O‒Si or Si‒C), and 1110–1130 cm^−1^ (asymmetric stretching vibration of Si‒O‒Si) [35,36].

In general, the FTIR spectra of RHs without and with pretreatment showed similar trend curves, suggesting that the leaching pretreatment does not change the functional group structure of SiO_2_ and the main organic matter (cellulose and lignin) in RH. However, the characteristic peaks of SiO_2_ (480 cm^−1^, 800–820 cm^−1^, and 1110–1130 cm^−1^), C=O group (1740 cm^−1^), and C‒H group (2929 cm^−1^) of RH samples by water- or acid-leaching pretreatment are more obvious than those of the unpretreated one. This is due to the fact that leaching pretreatment removes most of the metallic impurities, so that the concentration of SiO_2_ and carbon-containing compounds become more apparent, and the enhancing effect is more pronounced in the case of the acid-pretreated RH.

**Figure 4:**
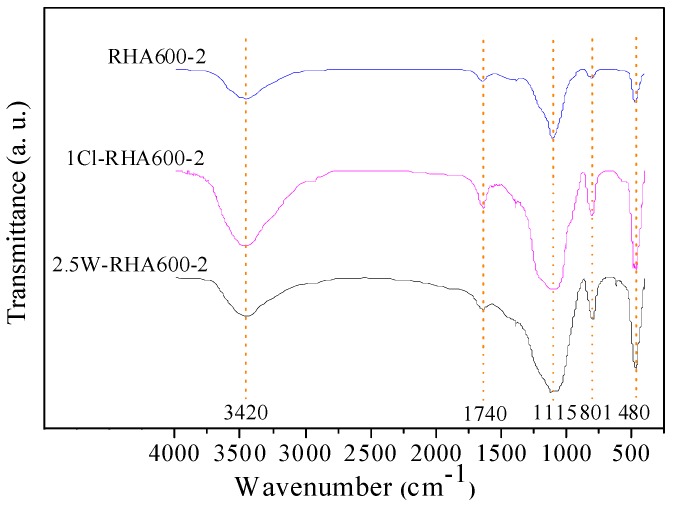
FTIR spectra of RHA with and without various pretreatments.

The FTIR spectra of RHAs are illustrated in Figure 4. It is seen that, after pyrolysis, the C‒H bond (2929 cm^−1^) and the C=O bond (1740 cm^−1^) referent to the functional groups of cellulose and lignin disappear for all the ash samples, suggesting that most of the organic compounds are burnt out. The intensity of SiO_2_ characteristic peaks at 480, 800–820, and 1110–1130 cm^−1^ of RHAs follows the ascending order of the untreated RHA, the water-leaching-pretreated RHA, and the acid-leaching-pretreated RHA, suggesting that the reactivity of the leaching-pretreated RHA is higher than that of the unpretreated one.

### 3.4. SEM Morphology of RHs

**Figure 5:**
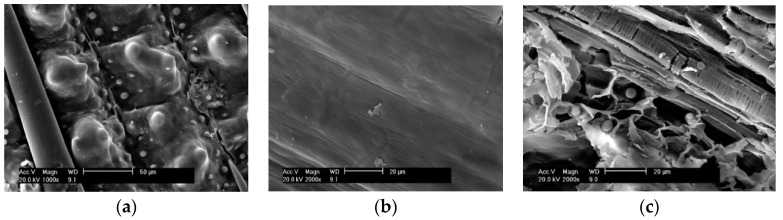
SEM morphologies of (**a**) outside surface; (**b**) inside surface and (**c**) interlayer of the water-leached RH.

The SEM morphologies of the outer surface, inside surface, and interlayer of the water-leaching-pretreated RH are presented in Figure 5. Figure 5a presents the outer surface of the water-pretreated RH. The outer surface is uneven and highly roughened. Many bulges are neatly arranged with a crochet sprout between. From Figure 5b, it is seen that the inner surface of the RH is smooth and dense. Figure 5c is a cross-sectional view of the RH interlayer. It is seen that the porous inside layer is a plate-like structure constituted of a multilayer mesoporous structure. It is apparent that the water-leaching pretreatment does not significantly change the surface morphology or inner skeleton structure of RH.

**Figure 6:**
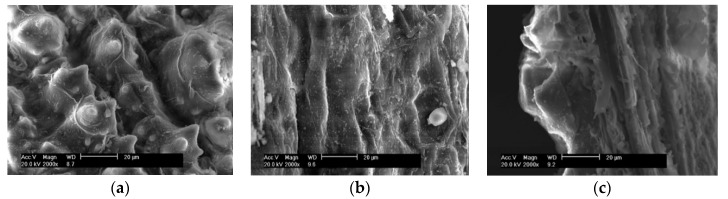
SEM morphologies of (**a**) outside surface, (**b**) inside surface, and (**c**) interlayer of the acid-leached RH.

The morphologies of the outer surface, inside surface, and cross section morphology of the HCl leaching pretreated RH are presented in Figure 6a–c respectively. It is seen that, after acid leaching, the outer surfaces, smooth inside surface, and porous interlayer of RH were significantly corroded as compared to the corresponding part of the water-leaching-pretreated one shown in Figure 5. These morphological changes are presumably due to the hydrolysis of some organic components by acid.

### 3.5. XRD Analysis of RHA

**Figure 7:**
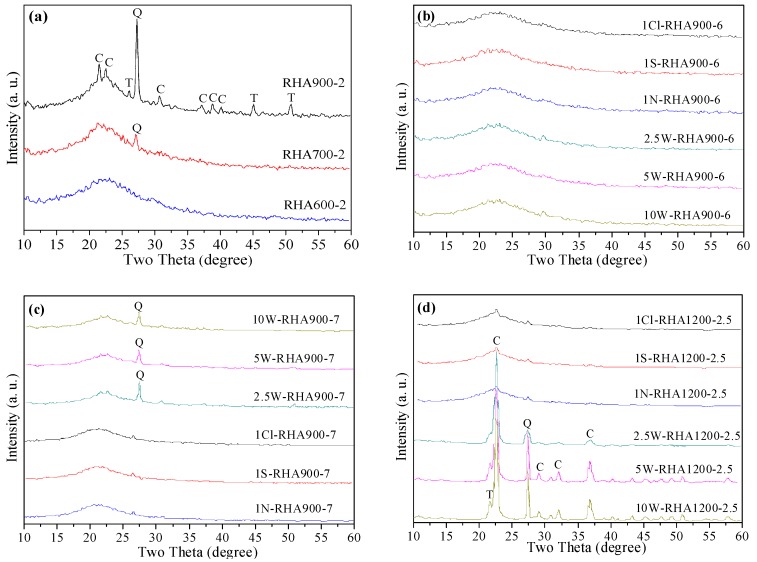
XRD spectra of RHAs obtained from: (**a**) unpretreated RHs burnt out at 600, 700, and 900 °C for 2 h; (**b**) various leaching-pretreated RHs burnt out at 900 °C for 6 h; (**c**) various leaching-pretreated RHs burnt out at 900 °C for 7 h; (**d**) various leaching-pretreated RHs burnt out at 1200 °C for 2.5 h. (C = cristobalite; Q = quartz; T = tridymite).

XRD spectra of the unpretreated RHA, the water-leaching-pretreated RHA, and the acid-leaching-pretreated RHA with various calcination temperatures and durations are shown in Figure 7. The XRD patterns of unpretreated RHA obtained by calcining at various temperatures are shown in Figure 7a. It is seen that broad diffused peaks with maximum intensity at 2*θ* = 22.5° are observed at RHA600-2, indicating the amorphous nature of silica. At 700 °C the presence of quartz (2*θ* = 26.7°) is observed. As the temperature increased to 900 °C, cristobalite (2*θ* = 22.5°) and tridymite (2*θ* = 26°) were also detected, as reflected by the peak intensities. These phenomena demonstrate the thermal instability of amorphous silica of unpretreated RH, which has been extensively shown in previous research works [5,37]. The phase transformation of RHA silica from amorphous to crystalline is very sensitive to pyrolysis temperatures above 700 °C. Hence, the crystallization sensitivity nature of RHA silica challenges the mass production of highly reactive silica based on existing industrial calcination equipment and techniques.

It has been reported elsewhere [38] that the presence of alkali metal salts results in a marked decrease in the crystallization temperature of the eutectic silica in RH. The calcination-temperature-dependent nature of silica is remarkably weakened by leaching treatment, as confirmed in Figure 7b. It is evident that acid-leaching-pretreated RHA silica shows completely amorphous structures upon combustion at 900 °C for 6 h. In addition, water-leaching-pretreated RHA silica presents similar mineralogical features at this thermal station. This is because of the removal of alkali metals, which preferably react with RHA silica to form eutectic composite with a low crystalline temperature. However, as the calcination duration extends to 7 h, the incipient cristobalite formation becomes apparent in the XRD patterns of the water-leaching-pretreated RHA silica powders. When pyrolysis takes place at 1200 °C for 0.5 h, the crystalline impurities cristobalite and quartz predominate. In the case of acid-leaching-pretreated RHA silica at this pyrolysis stage, tenuous peaks of cristobalite and tridymite gradually began to be visible, suggesting that crystalline silica formation starts to occur. It is seen that the ceiling capability for water-leaching pretreatment is to keep silica amorphous at 900 °C for 7 h. At 1200 °C, as shown in Figure 7d, small elevations corresponding to patterns of cristobalite and quartz arise in the acid-leached silica samples, i.e., acid-leaching pretreatment raises the crystalline transformation temperature of silica up to 1200 °C. In the case of water-leaching-pretreated RHA, cristobalite and quartz are formed to a large extent, along with the tenuous appearance of tridymite. It is apparent that the acid-leaching pretreatment has superior performance to the water-leaching pretreatment in reducing the crystallization sensitivity of RHA silica. However, the water-leaching treatment can use industrial cooling water as the leaching agent, hence reducing the investment in process technology and sophisticated equipment in comparison to acid-leaching treatment.

### 3.6. Influence of Alkali Metals on Crystallization in RHA Silica

**Figure 8:**
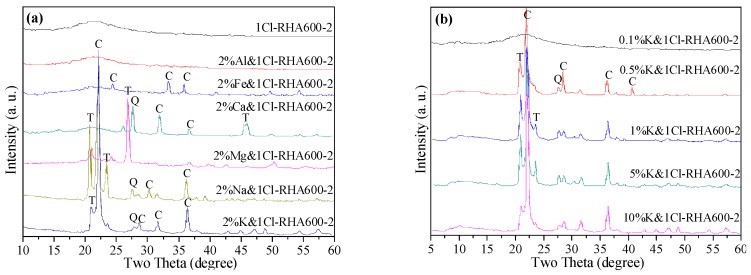
XRD spectra of (**a**) the mixture of HCl-pretreated RH and chlorine salts after burning out at 600 °C for 2 h; (**b**) the mixture of HCl-pretreated RHA silica various dosage of KCl after burning out at 600 °C for 2 h. (C = cristoballite; Q = quartz; T = tridymite).

To identify the influences of metallic impurity and content on thermal stability of RHA silica, mixtures of 1 N HCl-purified RH incorporated 2 wt % various alkali metallic trace elements in the form of chlorine salts are subjected to burning out at 600 °C for 2 h. The XRD spectra of the ash mixtures are plotted in Figure 8a. It is seen that a sharp intensity peak at 2*θ* = 22.5° corresponding to cristobalite is noticed in the XRD pattern. Similarly, the incorporation of NaCl_2_, MgCl_2_, and CaCl_2_ into silica promotes formation of the crystals cristobalite and quartz in the resulting blends. In the case of NaCl_2_ & 1Cl-RH ash blends, an incipient peak referent to the tridymite at 2*θ* = 23° is observed, suggesting that Mg promotes tridymite formation. However, silica blend with the incorporation of AlCl_3_ and FeCl_2_ shows no obvious SiO_2_ crystallization, indicating that the trace elements Al and Fe rarely contribute to promoting the crystallization of RHA silica.

Combined with the XRF chemical composition results, K is detected as the most abundant trace element in RHA, and hence it is the major contributor leading to the reduction of the crystallization temperature of the eutectic silica system. This finding is in line with the report of Krishnarao et al. [28]. It is also interesting to note the effect of K content on the crystallization behavior of RHA silica. The XRD spectra of the 1 N HCl-purified RH-incorporated mixtures with various addition ratios of K are given in Figure 8b. It is seen that a 0.1% addition of K has no impact on the crystallization of the silica blend. With the incorporation ratio of K increasing to 0.5% and above, the detraction crystalline peak corresponding to cristobalite becomes intensive and sharp. Thus, it is inferred that K exerts its function in accelerating the crystallization of RHA silica when its concentration in RHA is beyond 0.5%.

### 3.7. Compressive Strength of RHA Incorporated Cement Paste

**Figure 9:**
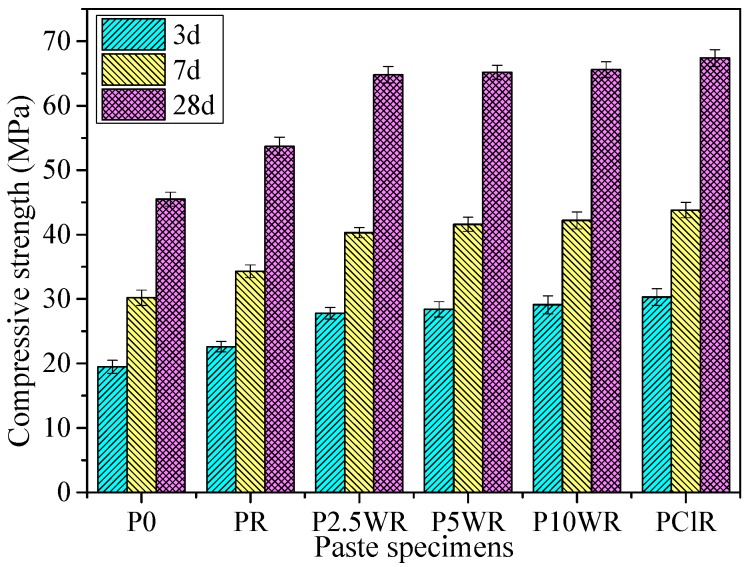
Compressive strength as a function of age for RHA cement paste.

To investigate the pozzolanic reactivity of the RHA silica, a compressive strength test of the blended cement paste was carried out. The compressive strength results of paste specimens at the age of three, seven, and 28 days are shown in Figure 9. P0 is the control paste. PR represents the paste with 10% cement replaced by the RHA obtained by burning RH at 600 °C for 2 h. P2.5WR, P5WR, and P10WR denote pastes with 10% cement replaced by the RHA obtained from water-leaching pretreatment on RH for 2.5, 5, and 10 h, respectively, and then burning out at 600 °C for 2 h. PClR stands for the paste with 10% cement replaced by the RHA obtained from 1 N hydrochloric on RH for 1 h and then burning out at 600 °C for 2 h.

It is seen that the compressive strength of paste undergoes a significant improvement when 10% of RHA is incorporated. The compressive strength of paste with unpretreated RHA is 20.2% higher than that of the control paste. The enhancing effect of RHA is improved by leaching pretreatment on RH prior to calcination. This can be attributed to the high silica content and high specific surface area of the leaching-pretreated RHA silica. The highest compressive strength appears in the paste with incorporation of acid-leaching-pretreated RHA (67.4 MPa), and the value is 48.1% higher than that of the control paste. In the case of the water-leaching-pretreated, RHA-incorporated cement paste, the RH with water-leaching pretreatment for 2.5 h yields the resulting RHA-blended paste having a compressive strength of 64.8 MPa, and this value is 42.4% higher than that of the control paste. Prolonging the water-leaching duration to 10 h, the strength value of the resulting ash-incorporated cement paste is increased to 65.6 Mpa. It is apparent that extending the water-leaching duration does not lead to obvious improvement in the pozzolanic reactivity of RHA. Thus, 2.5 h is an acceptable duration for water-leaching pretreatment on RH to yield a highly reactive pozzolana. It is worth noting that the water-leaching-pretreated RHA has better performance at improving the compressive strength of cement paste as compared to undispersed silica fume, as reported by Ji et al. [39], and presents comparable pozzolanic reactivity to dispersed silica fume and metakaolin, as per [40]. The enhancing effect of RHA on the compactness of the cement body is attributed to the silicate tetrahedron of RHA participating in the composition of hydrated calcium silicate (C‒S‒H) to prolong the chains of C‒S‒H, and soluble silicate‒oxide chains of RHA also react with the free Ca(OH)_2_ released from the cement hydration to form more C‒S‒H phase in the cement matrix [41].

### 3.8. Chemically Bound Water Content of RHA Incorporated Cement Paste

To investigate the effect of the RHA silica on the hydration degree of cement matrix, the chemically bound water of the paste fragments of the above compressive strength test was calculated. 

The water in the hardened cement paste is divided into evaporated water (free water) and non-evaporated water (chemically bound water). A study [42] has revealed that the volume of evaporating water can be used as a measure of the pore volume in the cement paste, while the amount of non-evaporating water refers to the amount of hydration products. Therefore, the amount of chemically bound water measured at different ages can be used as a representative value of the degree of hydration of cement paste. The unit mass of chemical combined water was calculated as in the following equation:

X1 = (M1 − M2)/M2 − Xad.c/(1 − Xad.c), (1)

where X1 is the chemical combined water content per unit mass of cementitious material, M1 is the quality of the paste sample after drying at 105 °C, and M2 is the quality of the sample after drying at 1000 °C. Xad.c refers to Equation (2):

Xad.c = XadXad.1 + XcXc.1,(2)

where Xad and Xc are the mass fraction of the admixture and cement, respectively; Xad.1 and Xc.1 are the loss on ignition of the admixture and cement, respectively.

The amount of chemically bound water of the cement paste at the early age is expressed in terms of unit mass of cement and denoted as X2, as per Equation (3):

X2 = X1/(1 − Xad).(3)

To quantitatively characterize the effect of admixture on cement hydration, a hydration influence factor F of the admixture is introduced and calculated as F = equivalent bound water (X2)/combined water of the pure cement at the same age. If F is more than 1, the admixture promotes the hydration of the cement. The larger the value, the more obvious the promotion effect; if F is less than 1, the admixture delays the hydration of the cement. The larger the value, the more obvious the delay effect.

The chemically bound water results of RHA incorporated cement paste specimens are shown in Table 3. It is seen that the chemically bound water in control paste was only 11.3% and 19.7% at the age of and 28 days, while addition of the RHA results in a remarkable increase in the content of chemically bound water in the resulting paste at each age. In the case of the paste with incorporation of the water-leaching-pretreated RHA, the chemically bound water of 28 days is improved to 29.3 to 30.2% depending on the leaching duration, and the variation is not very noticeable. The paste with hydrochloric acid pretreated RHA shows the chemically bound water content is 30.3%, which is the highest value. These calculation results are in line with the conductivity and compressive strength results. It is also worth noting that the boiling-water-pretreated RHA silica has a comparable performance at improving the cement hydration with the acid-pretreated one.

### 3.9. XRD Analysis of Hydration Products of RHA Incorporated Cement Paste

**Figure 10:**
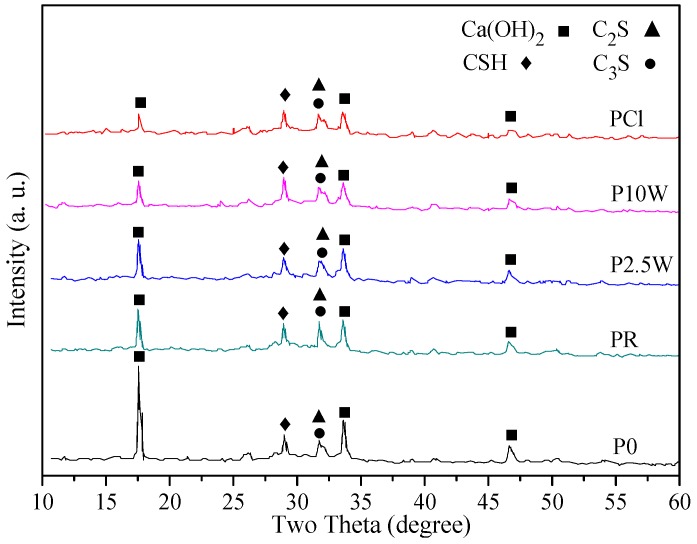
XRD patterns of RHA cement paste at the age of seven days.

To investigate the effect of RHA silica on the hydration products of cement, the XRD analysis of paste samples P0, PR, P2.5W, P10W and PCl were determined at the age of 7 days. The results are plotted in Figure 10. It is seen that the intensity of Ca(OH)_2_ diffraction peak in the control cement paste P0 is very sharp. The addition of RHA weakens the X-ray diffraction peak intensity of Ca(OH)_2_. The phenomenon is attributed to the fact that silica in RHA can react with the free Ca(OH)_2_ to form crystalline or semi-crystalline hydrated calcium silicate (CSH), improving the compactness of the hardened cement body. In addition, the intensity of Ca(OH)_2_ diffraction peak is in the ascending order of the paste PCl, P10W, P2.5W, PR, and P0, indicating the pozzolanic reactivity of RHA following the descending order of 1Cl-RHA600-2, 10W-RHA900-2, 2.5W-RHA900-2, and RHA600-2. These results are in line with the compressive strength test and chemically bound water content test results.

## 4. Conclusions

After burning, RHs become RHA that normally contained 90% silica (SiO_2_), 5–8% of alkali metal oxides, and some carbonaceous materials by mass. The reactive amorphous silica is extracted from RH via burning out of organic cellulose and lignin under controlled calcination at 600 °C for 2 h. Raising the calcination temperature or extending the calcination duration can increase the silica concentration but lead to accelerated formation of crystalline compounds (mainly in the form of quartz and cristobalite) in the ashes. Potassium was proven to cause surface melting of a eutectic potassium‒silica compound, restraining carbon emissions from burning RH and hence leading to the formation of fixed black carbonaceous matter and accelerating the crystallization of amorphous silica to form cristobalite.

Acid-leaching pretreatment can effectively remove the concentration of alkali metal impurities in RH. An amorphous silica with purity above 96% is yielded from RH by 1 N hydrochloric acid leaching followed by calcination at 600 °C for 2 h. Sulfuric acid and nitric acid have comparable rinsing performance to hydrochloric acid. The acid treatment increases the crystallization temperature of RHA silica to 1200 °C and retains the amorphous state for 2.5 h.

Water-leaching pretreatment also exerted sound performance in rinsing off the alkali metal impurities. A reactive amorphous silica with a concentration of 94% is yielded via water-leaching pretreatment for 2.5 h and pyrolysis of 600 °C for 2 h. Moreover, water-leaching pretreatment on RH for 2.5 h could maintain the amorphous state of silica at 900 °C for 7 h.

The compressive strength of the acid-leaching-pretreated, RHA-incorporated paste is 48.1% higher than that of the control paste. The RH with boiling-water-leaching pretreatment for 2.5 h yields ash-blended paste having a compressive strength of 64.8 MPa, which was 42.4% higher than that of the control paste. Expanding the water-leaching duration did not obviously improve the pozzolanic reactivity of RHA. Thus, 2.5 h was an acceptable duration for boiling-water-leaching treatment on RH to achieve a highly reactive pozzolana. In addition, the boiling-water-leaching-pretreated RHA silica has a comparable pozzolanic performance at improving the cement hydration to the acid-pretreated one, as reflected by the calculation results of chemically bound water content.

In summary, the boiling-water-leaching pretreatment is slightly inferior to the hydrochloric-acid-leaching pretreatment at improving the thermal stability and removing the metal impurities of RHA silica. However, the boiling-water-leaching treatment is superior to the acid-leaching pretreatment in using an industrial cooling water system and hence reducing the investment in process technology and sophisticated equipment. Furthermore, the amorphous silica obtained under the water-leaching‒pyrolysis treatment achieves a biomass amorphous silica with high purity and large surface area, and hence can be produced at a large scale as a highly reactive pozzolana for applying in the production of high-strength cement and concrete. Thus, this study is of great significance for the extension of RHA in the practical application of cement engineering.

## Figures and Tables

**Table 1:** Chemical composition of RHAs.

RHA Samples	SiO_2_ (%)	K_2_O (%)	Na_2_O (%)	CaO (%)	MgO (%)	Al_2_O_3_ (%)	Fe_2_O_3_ (%)	LOI (%)
RHA600-2	89.61	2.53	0.16	1.52	0.56	0.36	0.90	3.53
RHA600-8	90.42	2.48	0.14	1.57	0.59	0.39	0.88	3.74
RHA900-2	91.76	2.13	0.11	1.33	0.62	0.33	0.79	4.61
RHA1200-0.5	92.23	1.92	0.09	1.21	0.71	0.28	0.37	4.92
1Cl-RHA600-2	96.41	0.06	0.05	0.07	0.24	0.19	0.28	1.80
1S-RHA600-2	95.52	0.07	0.07	0.64	0.22	0.21	0.32	2.24
1N-RHA600-2	95.81	0.06	0.06	0.59	0.22	0.18	0.29	2.11
2.5W-RHA600-2	94.03	0.26	0.12	0.95	0.32	0.23	0.33	3.32
2.5W-RHA900-2	94.42	0.24	0.09	0.99	0.41	0.22	0.31	2.04
5W-RHA900-2	94.80	0.22	0.10	0.95	0.29	0.21	0.29	2.96
10W-RHA900-2	94.92	0.21	0.11	0.92	0.29	0.19	0.28	2.90

Note: LOI represents the loss of ignition.

**Table 2:** Particle size, BET surface area, and conductivity of RHAs.

RHA Samples	Average Particle Size (μm)	BET Surface Area (m^2^/g)	Total Pore Volume (cm^3^/g)	Average Pore Diameter (nm)	Conductivity Change (ms/cm)
RHA600-2	5.47	74.88	0.082997	32.78	0.38
RHA600-8	6.21	68.83	0.072315	33.19	0.42
RHA900-2	6.47	10.59	0.053497	35.14	0.11
RHA1200-0.5	6.96	10.02	0.042749	38.17	0.00
1Cl-RHA600-2	5.02	248.21	0.368958	5.15	6.09
1Cl-RHA900-2	5.63	250.32	0.312846	6.07	6.11
1Cl-RHA1200-0.5	6.21	200.02	0.287963	7.70	0.22
1S-RHA600-2	5.20	232.97	0.324875	5.38	5.73
1N-RHA600-2	5.14	241.76	0.357947	5.23	5.91
2.5W-RHA600-2	5.34	130.82	0.257617	6.45	4.42
2.5W-RHA900-2	6.02	120.85	0.184237	7.02	4.64
5W-RHA900-2	5.88	133.78	0.274633	6.88	4.75
10W-RHA900-2	5.42	137.96	0.302141	6.64	4.87

**Table 3:** Chemical combined water and factors of RHA pastes.

Paste Samples	7 Days		28 Days	
	X1	F	X1	F
Control	11.3%	1	19.7%	1
PR	11.8%	1.16	21.6%	1.22
P2.5WR	18.0%	1.77	29.3%	1.65
P5WR	18.5%	1.82	29.5%	1.66
P10WR	18.7%	1.84	30.2%	1.70
PClR	18.7%	1.86	30.3%	1.72
